# Trauma: a major cause of death among surgical inpatients of a Nigerian tertiary hospital

**DOI:** 10.11604/pamj.2017.28.6.10690

**Published:** 2017-09-05

**Authors:** Onyeanunam Ngozi Ekeke, Kelechi Emmanuel Okonta

**Affiliations:** 1 Department of Surgery, University of Port Harcourt Teaching Hospital, Alakahia, Port Harcourt, Nigeria

**Keywords:** Trauma, mortality, surgical wards, Port Harcourt, Nigeria

## Abstract

**Introduction:**

Trauma presents a significant global health burden. Death resulting from trauma remains high in low income countries despite a steady decrease in developed countries. Analysis of the pattern of death will enable intervention to reduce these deaths from trauma in developing countries. This study aims to present the pattern of trauma-related deaths in the surgical wards of University of Port Harcourt Teaching Hospital (UPTH).

**Methods:**

This was a retrospective study of all patients who died from trauma during admission into the surgical wards of UPTH from 2007 to 2012. Data on demography and traumatic events leading to death were collected from surgical wards, the emergency unit, and theatre records and analyzed using SPSS version 16.0.

**Results:**

Trauma accounted for 219 (42.4%) of the 527 mortalities recorded. Most of the deaths (62.6 %) occurred between 20 and 59 years. There were 148 males (67.6 %). The yearly mortality rates were as follows: 2007(12.3 %); 2008 (16.9%); 2009 (9.1%), 2010 (12.8 %), 2011 (23.3%) and 2012 (25.6%). Most of the patients (91.3%) died within 1 month of admission. The major events leading to deaths were burns 105(47.9%), traumatic brain injuries were 63(28.8%), and spinal cord injuries 21(9.6%). The secondary causes of death were mainly septic shock 112(51.1%); Respiratory failure 60(27.4%); and Multiple organ dysfunction 44(20.1%).

**Conclusion:**

Trauma is a leading cause of mortality in the surgical wards of our hospital. Trauma -related deaths continues to increase over the years. Safe keeping of petroleum products and adherence to traffic rules will reduce these avoidable deaths.

## Introduction

Trauma is a physical harm from the external environment causing damage to a biological organism. It may eventually lead to death. It may result from road traffic accidents, burns, falls, gunshot injuries, electrical currents, etc. The burden of injuries worldwide is disproportionately concentrated in low- and middle-income countries (LMIC) [1]. According to the World Health Organization, over 91% of unintentional injury deaths and 94% of disability-adjusted life-years were lost in LMIC in 2004 [2,3]. Unintentional injuries accounted for over 7% of total deaths and over 9% of total disability-adjusted life-years in these countries. The highest injury burden often occurs in those countries with the weakest evidence to guide intervention strategies, the fewest resources, and the least developed infrastructure to effect change. Injury is now the fourth leading cause of global deaths, and up to 2030 World Health Organisation (WHO) estimates a further 40% increase in trauma fatalities [3].

Trauma predominantly occurs in the young adult males with road traffic accident being the leading aetiological factor [4,5]. Most deaths following trauma are associated with multiple injuries (60.9%) and head injuries (30.4%) [6]. The most frequently injured region of the body noted wasthe head and neck (53.4%) in Benin City [5]. In the same vein, burn injuries remain a global problem even though they are largely preventable. Burn injuries continue to be a major source of mortality and morbidity in low- and middle-income countries of the world, of which Nigeria is a part [7-9]. The patterns of mortality vary widely by cause, age, sex, region and time [10]. Painful though, most of these trauma-related deaths have not been carefully identified even asthey are preventable [7]. Alas,incomplete documentation of accident and injury data occurred frequently [6]. Identification of the factors responsible for trauma related deaths in the surgical wards of an institution will help in appropriate allocation of resources in resource limited countries. This study aims to identify the causes of trauma death in the surgical units of UPTH.

## Methods

This was a retrospective study of all the mortality recorded from surgical admission following trauma in the University of Port Harcourt Teaching Hospital from 2007 to 2012. UPTH is an 800 bed tertiary hospital that receives referrals from neighbouring states-Abia, Bayelsa, Delta, Imo, Akwa Ibom states. The data were collected from admissions into the surgical wards of UPTH from the accident and emergency unit, surgical wards, theatre, intensive care units and medical records department. The case notes of the patients who died while on admission in the surgical wards were retrieved and the cause of death recorded. Those whose primary causes of death were from trauma were further isolated and entered into a proforma. Information on their biodata, year of death, duration of admission, diagnoses, primary cause of death ,secondary cause of death and the respective surgical units were collected and analyzed using computer based statistical software SPSS version16.0, (IBM, Chicago, Illinois). The primary cause of death was defined as the organ/system involved in trauma that started the cascade of events leading to death. Secondary cause of death was considered as the complications and other events that may have contributed to the death.

## Results

**Figure 1:**
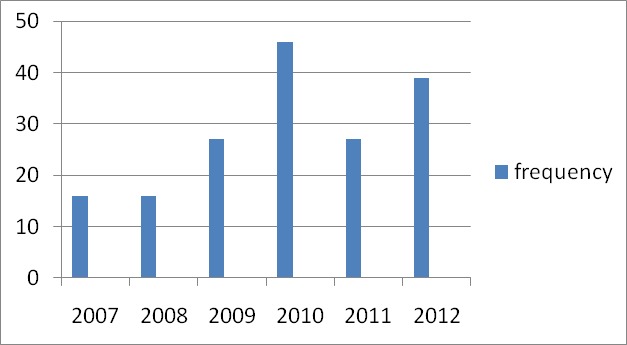
Mortality per year

Two hundred and nineteen trauma-related deaths (42.4%) from a total 527 mortalities recorded from the 8230 patients admitted during the 6- year period giving a mortality rate of 2.1%. Most of the deaths (62.6 %) occur between 20 and 59 years. The age distribution is shown in Table 1. One hundred and forty-eight deaths (67.6 %) occurred in males while 71(32.8%) were females. The yearly mortality rate was as follows: 2007(12.3 %); 2008(16.9%); 2009(9.1%); 2010(12.8 %), 2011(23.3%) and 2012(25.6%) as shown in Figure 1. When the units were considered, the mortality rates were as follows: the General surgery unit 6(2.7%); Burn and plastic unit 104(47.5 %); Neurosurgery unit 62 (28.3 %); Urology unit 1(0.5%); Orthopaedics unit 42(19.2 %), Paediatric surgery unit 3 (1.4 %), and cardiothoracic surgery unit 1(0.5%). The distribution of the deaths per unit is shown in Table 2.

**Table 1:** Age distribution

Age Range (Years)	Frequency (n)	Percent (%)
0-9	24	11.0
10-19	19	8.7
20-29	58	26.5
30-39	39	17.8
40-49	25	11.4
50-59	15	6.8
60-69	19	8.7
70-79	10	4.6
>80	10	4.6
TOTAL	219	100.0

**Table 2:** Death from different specialties

Specialty	Frequency (n)	Percent (%)
Burns and plastics	104	47.5
Cardiothoracic	1	0.5
General surgery	6	2.7
Neurosurgery	62	28.3
Orthopaedics	42	19.2
Paediatrics	3	1.4
Urology	1	0.5
TOTAL	219	100.0

When the cause of death was considered; Patients with burns were 105(47.9%), patients with traumatic brain injury were 63(28.8%), spinal cord injury 21(9.6%) patients with fracture of long bones were 13(5.9%), penetrating abdominal injuries 6(2.7%), multiple injuries 5(2.3%), foot gangrene 3(1.4%); chest trauma 1(0.5%). The primary causes of deaths were illustrated in Table 3. Most of the patients (91.3%) died within 1 month of admission. The duration of admission is shown in Table 4. The secondary causes of death were as follows: septic shock 112(51.1%), respiratory failure 60(27.4%), multiple organ dysfunctions 44(20.1%), haemorrhage 2(0.9%), acute kidney injury 1(0.5%).

**Table 3:** Primary cause of deaths

Primary cause	Frequency (n)	Percent (%)
Burns	105	47.9
Traumatic Brain Injury(TBI)	63	28.8
Fractures	13	5.9
Spinal cord Injury	21	9.6
Pelvic fracture	2	0.9
Multiply Injured	5	2.3
Penetrating Abdominal Injury	6	2.7
Others (Haemothorax, Foot gangrene etc)	4	1.8
TOTAL	219	100.0

**Table 4:** Duration of admission before death

Duration (days)	Frequency (n)	Percent (%)
0-28	200	91.3
29-56	7	3.2
57-84	6	2.7
85-112	3	1.4
113-140	1	0.5
141-168	0	0.0
169-196	1	0.5
197-224	0	0.0
225-262	1	0.5
TOTAL	219	100.0

## Discussion

Trauma is one of the leading causes of death in most countries [5,11,12]. Little has been done to curb tribal fighting and domestic violence. Road traffic fatalities have at least remained static in the last decade and wearing seat belts is now compulsory, but enforcement of the law is still challenging. Driving after drinking alcohol is another contributory factor to road traffic accidents [13]. Trauma contributed to almost half of the deaths in our surgical wards. This was similar to the 38.8% reported by Ayoade as the leading cause of death in Sagamu [14]. The high rate of trauma-related deaths also corresponds to high rate of trauma-related admissions which as was previously reported in Kenya, another low income country where 73.5% surgical admissions were for trauma [15].

The reasons for the high mortality rate in this study may be due to neglect of safety rules on driving. A lot of the people, who ride motor cycles, do so without wearing protective gears. Bad roads increase road traffic accidents. Election-related violence, thuggery, cultism,armed robbery, and kidnappings, and militancy are major causes of injury in our city. Traffic congestion is another major challenge in this city. Traffic jams impede the smooth movement of casualties to centres where they can be treated. There is also a dearth of ambulance services and critical care units, thus the patients often arrive the hospital late and in very critical conditions. A lot of people in our city lack the knowledge of first aid. Therefore, some of the patients are transported in ways that often jeopardize their medical conditions. Most of the patients don’t get the appropriate care before arriving the hospital; and they may therefore die from the injuries that may be salvageable in more developed countries.

While Trauma-related deaths continue to be high in the developing countries, the reverse is the case for developed countries [16]. In Germany, there was a decrease by 68.8% from 1991-2011.The decrease of severe injuries after road traffic accidents was attributed to a comprehensive approach including the enforcement of road safety policies and innovations in car engineering and emergency medicine. Traffic related measures, alcohol level control, seat belt usage enforcement as well as other technical advances were considered especially important [16]. Most deaths in this study occurred in young men. This is similar to a report in Kenya where males constituted 73.5% of all trauma admissions and 57.6% were in the 21-60 year age-group [15]. Trauma predominantly occurs in the young adult male with road traffic accidents being the leading aetiological factor [4,5]. The young adults are very energetic and mobile. They are more likely to engage in civilian violence. This is the age group that are involved in close combats, militant and armed robbery attacks, and youth restiveness in the Niger Delta region of Nigeria [17]. This may explain the predominance of young males in this study.

Injuries continue to be an important cause of morbidity and mortality in the developed and developing world. The decline in rates for almost all injuries is so prominent that it warrants a general statement that the world is becoming a safer place to live in [10]. This statement is not true in many developing countries like ours.Reports in Tanzania confirm increasing incidents of injuries over the years [18]. In our study, there was steady increase in the number of cases every year except in 2009 when our hospital did not have many admissions due to industrial actions by staff. Perhaps the trauma related deaths could even have been more than what it was in this study but for the ban on commercial usage of motor cycles as means of intra-city transport.

Burn injuries are one of the aetiologies of trauma and are the major cause of prolonged hospital stays, disability, and death in Africa [19]. From this study, burns injuries were the major causes of death accounting for over half of the deaths recorded. The reasons for this high mortality from burn injuries abound: Port-Harcourt is one of the major petrol producing cities in the country and thus prone to explosive disasters occurring in oil-producing countries like Nigeria [8]. This has been evident even from previous review about 3 decades ago that showed that Petrol fire was the commonest cause of flame burns and alas still remains so up till now [20]. Burn mortality occur when the burn surface area in a patients is over 50 per cent of the total body surface area [20] and this is still our experience till date as facilities and the expertise to manage this degree of burn is suboptimal in our center. Burns tend to increase in places where there is paucity of burn safety knowledge [7]. High levels of formal education correspond to a higher degree of burn safety knowledge [7]. It can be generally noted from this study that in spite of all that has been said about this condition there had not been strong actions toward the prevention, pre-hospital management and the actual treatment of burn in Port-Harcourt city. Thus, the carnage occasioned by this condition continues un-abated. People still go to scoop fuel from fallen petrol carrying tankers. Sometimes, the spilled fuel will be ignited burning the scoopers.

Another important cause of increased trauma death in our environment was traumatic brain injuries managed by the neurosurgical unit in UPTH. Traumatic Brain Injuries resulting from trauma predominantly affects young male population and most of these are preventable [11]. A study done in this city about a decade ago showed that the head was the commonest body region affected in trauma- related deaths [21]. Though that study was in paediatric age group, the pattern is similar to the observation in this study. Also, in another study that specifically considered patients who died from road traffic crash, intracranial hemorrhage was the predominant cause of death [22]. There is plethora of reasons for the high death coming from head injuries following trauma. Most of the motorcycle riders do not put on helmets while riding their motorbike in Port Harcourt city. This picture is common in Nigeria [23,24]. Motorcycle helmets reduce the risk of death and head injury in motorcycle riders who crash [25]. The lack of use of seat belts predisposed patients to sustaining head injuries when they are involved in road traffic crash [26]. The challenges of managing severely head injured patients are in the provision of adequate Intensive Care Unit services with elaborate investigations with Computerized Tomography scan. These are currently not adequate in UPTH. A lot of our patients present first to health facilities that lack the expertise for head injuries management before presenting in UPTH. Improvement in head trauma care will entail early transfers of patients to appropriately equipped health care facilities for initial resuscitation and appropriate surgical management [27]. The predictors of mortality in patients with fractures such pelvic fractures are middle aged, geriatric, injury severity among other reasons [28]. In our observation, most of these patients were multipled injured with high injury severity. The accompanying complications were mainly sepsis and haemorrhage.

The explanation for low mortality recorded in chest injuries was as a result of the great deal of understanding that in most cases of chest injuries with complications of pleural collections, the prompt institution of chest tube was all that may be required in most cases to avoid death [29]. Generally, the education of accident and emergency department care providers in basic principles of stabilization and initial treatment are most cost-effective method of reducing preventable deaths [30].

It has been reported that 67.4% of the deaths occurred within six hours of presentation to an accident and emergency unit [5]. Almost all the deaths were recorded in this study occurred within the first 28 days and are due to multiple organs failure and infection. Other reasons noted by previous observers especially the ones occurring either within the first week of injury are inappropriate pre-hospital treatment, pre-hospital delays and inappropriate mode of transportation without inter-hospital communication [27]. However, in our own case, beyond the previously stated reason expertise and appropriate facilities were sometimes lacking.

## Conclusion

Trauma is a major contributor to mortality among our surgical inpatients. Policies on injury prevention that have been promulgated are yet to be fully complied with. It appears that previous suggestions on the prevention of trauma in our setting have not been backed up with commiserating actions thus trauma from various causes still subsist. Trauma as a major cause of mortality can be prevented by ensuring mandatory training in trauma management, safe method of storing petroleum product, wearing of helmets while riding motorcycle and apportioning of reasonable resources to tackle them. Improvement of infrastructure and road maintenance, peaceful resolution of conflicts as well as political education and moral instructions will reduce civilian violence. Employment and positive engagements of youths will reduce youth restiveness which will ultimately reduce trauma related deaths. Improvement on hospital equipment and training of personnel will reduce death among the injured.

### What is known about this topic

- Trauma is recognized cause of mortality worldwide;
- Young adults are mainly affected;
- Trauma-related mortality is decreasing in developed countries.

### What this study adds

- Mortality from trauma is unacceptably high in our sub-region;
- Youth restiveness, militancy, political violence, cultism, thuggery poor infrastructure and suboptimal health care, are major contributors to these deaths;
- Previously proposed preventive measures are yet to be fully implemented.

## Competing interests

The authors declare no competing interests.
